# A Sensitive Liquid Chromatography-Tandem Mass Spectrometry Method for the Determination of Nimbolide in Mouse Serum: Application to a Preclinical Pharmacokinetics Study

**DOI:** 10.3390/pharmaceutics10030123

**Published:** 2018-08-08

**Authors:** Lingzhi Wang, Do-Dang Khoa Phan, Nicholas Syn, Xiaoqiang Xiang, Hongyan Song, Win Lwin Thuya, Shili Yang, Andrea Li-Ann Wong, Alan Prem Kumar, Wei Peng Yong, Gautam Sethi, Paul Chi-Lui Ho, Boon Cher Goh

**Affiliations:** 1 Cancer Science Institute of Singapore, National University of Singapore, Singapore 117599, Singapore; khoaphan@u.nus.edu (D.-D.K.P.); nicholassyn@gmail.com (N.S.); csithuya@nus.edu.sg (W.L.T.); csiawla@nus.edu.sg (A.L.-A.W.); csiapk@nus.edu.sg (A.P.K.); wei_peng_yong@nuhs.edu.sg (W.P.Y.); phcgbc@nus.edu.sg (B.C.G.); 2 Department of Pharmacology, Yong Loo Lin School of Medicine, National University of Singapore, Singapore 117600, Singapore; 3 Department of Pharmacy, National University of Singapore, Singapore 117543, Singapore; lsiysh@nus.edu.sg (S.Y.); paul.ho@nus.edu.sg (P.C.-L.H.); 4 Department of Haematology & Oncology, National University Cancer Institute, National University Health System, Singapore 119082, Singapore; 5 Department of Clinical Pharmacy, School of Pharmacy, Fudan University, Shanghai 201203, China; xiangxq@fudan.edu.cn; 6 Institute of Materials Research and Engineering (IMRE), ASTAR, Singapore 138634, Singapore; songhy@imre.a-star.edu.sg

**Keywords:** nimbolide, LC-MS/MS, mouse, serum, pharmacokinetics

## Abstract

A sensitive and robust liquid chromatography-tandem mass spectrometric (LC-MS/MS) method was developed and validated for the determination of nimbolide in mouse serum. Exemestane was used as the internal standard (IS). Here, we employed acetonitrile-based protein precipitation (PPT) for serum sample preparation, and performed chromatographic separation using an ODS Hypersil C18 column (100 mm × 2.1 mm, 5 µm) with gradient elution (0.1% formic acid in water vs 100% acetonitrile). The run time was 6 min. Instrumental analysis was performed by electrospray ionization tandem mass spectrometry (ESI-MS/MS) in the multiple-reaction monitoring (MRM) under positive mode. A good linear calibration was achieved in the 5–1000 ng/mL range. The intra- and inter-day precisions for nimbolide were ≤12.6% and ≤13.9% respectively. Intra-day accuracy ranged from 96.9–109.3%, while inter-day accuracy ranged from 94.3–110.2%. The matrix effect of nimbolide, detected but consistent at low and high concentrations, do not affect linearity of standard curve. In conclusion, we have developed and validated a sensitive analytical method for determination of a novel natural compound nimbolide in mouse serum, and it has been successfully applied to our preclinical study in investigating the pharmacokinetic properties of nimbolide, which could greatly facilitate the preclinical development of the promising lead compound for anticancer therapy.

## 1. Introduction

Drug development is traditionally a notoriously time-consuming and costly process involving multiple stages. Specifically, these stages include target identification, target validation, lead generation, as well as optimization, preclinical pharmacology, and finally clinical trials. Pharmacokinetics (PK) plays a crucial role throughout the drug development pipeline, including preclinical pharmacological investigations and Phase I/II clinical trials [1]. In the past, the attrition rate of up to 40% of new investigational agents could be attributed to poor PK profiles of early lead compounds [2].

This situation has dramatically changed, however, owing to advances in the development of liquid chromatography-tandem mass spectrometric (LC-MS/MS) methods, which enabled sensitive and specific quantification of drugs in various biological matrixes such as serum, plasma and tissue. Importantly, these approaches allow for rapid identification of PK constraints that may hinder future development of new drug candidates, thereby allowing appropriate remedial actions to be rapidly taken in the early drug development process. However, as progress was made in the sensitivity and specificity of the LC-MS/MS assays, it became increasingly apparent that the robustness of these assays hinged on the suitability of the internal standard (IS) reagent. The IS is needed to correct for variations in mass detection signals which may arise from various sources, including sample preparation steps, as well as chromatographic system-derived errors with regard to injection volume, detector response, pump, flow rate and column homogeneity as for routine HPLC assays [3].

However, currently employed MS/MS detectors have been found to be less stable and robust compared with conventional UV and fluorescence detectors, despite their superior sensitivity and specificity. Moreover, the co-elution of endogenous compounds has been shown to alter the ionization of the analyte in unpredictable ways [4]. Hence, in theory, an IS with comparable or proportional hydrophobicity and ionization as an analyte of interest may be used to improve the quality of MS/MS assays via compensating the errors (e.g. isotopically-labelled internal standards). However, the isotopically-labelled internal standards are very costly, and not commercially available for most of analytes. Hence, chemical analogues, or even non-analogues, are usually adopted as an internal standard instead for novel compounds discovered in natural resource. Despite the importance of IS in determining the accuracy of the concentrations of an analyte in biological matrices, principles guiding the selection of a suitable IS in LC-MS/MS method development and validation remain largely undefined. 

Nimbolide, a tetranortriterpenoid isolated from the leaves and flowers of the neem tree (*Azadirachta indica*), exhibits a variety of therapeutically-valuable properties, including having anti-malarial, anti-bacterial, anti-feedant and antioxidant actions [5,6,7,8,9,10,11]. Existing analytical approaches for quantitative determination of nimbolide include HPLC and High Performance Thin-Layer Chromatography (HPTLC)-based methods [12,13]. Both methods have been developed for the quantification of nimbolide in different parts of the *Azadirachta indica* plant and its dosage form. However, the sensitivity and specificity of existing methods do not meet the requirement of PK studies of nimbolide in biological matrices.

In this study, we used a non-analogue IS, exemetane, to develop a sensitive and robust bioanalytical method for the quantitative analysis of nimbolide in accordance with US Food and Drug Administration (FDA) guidelines [14], and applied the validated method to a preclinical PK study of this agent in mice.

## 2. Materials and Methods

### 2.1. Chemicals and Reagents

Nimbolide (the reference standard), exemestane, and paclitaxel were purchased from Toronto Research Chemicals Inc. (North York, ON, Canada). Methanol, acetonitrile, and formic acid (100%, *v*/*v*) were purchased from Merck (Darmstadt, Germany). Milli-Q water from Milli-Q Plus system (Millipore, Milford, MA, USA) was used throughout this study. Drug-free blank mouse plasma was obtained from mice bred in National Cancer Centre of Singapore.

### 2.2. Stock Solutions, Calibration Standards, and Quality Control Samples

Nimbolide is sparingly soluble in water, but soluble in organic solvents such as DMSO and MeOH. Hence, stock solutions of nimbolide and exemestane were prepared in methanol at 1.0 mg/mL. Six standard working solutions of nimbolide were prepared by serial dilution with methanol to attain concentrations of 5, 25, 100, 250, 500, and 1000 ng/mL. Working solutions of exemestane and paclitaxel at 250 ng/mL were prepared for the selection of a suitable IS. Three quality control (QC) working solutions of nimbolide were prepared by serial dilution with methanol to attain concentrations of 15, 300 and 900 ng/mL. All stock and working solutions were stored in the refrigerator at 4 °C.

### 2.3. Calibrator and Quality Control Sample Preparation

An aliquot of blank mouse plasma (10 μL) was placed into a 1.5 mL polypropylene (PP) centrifuge tube, followed by the addition of 10 μL of standard working solution and 10 μL of the IS working solution respectively. The PP tube was vortexed for 30 s after the addition of 30 μL of acetonitrile. The PP tube was centrifuged at 17,562× *g* for 10 min at 4 °C. Thereafter, 40 μL of the supernatant was transferred to a second 1.5 mL PP tube containing 60 μL of 0.1% formic acid in water. The tube was vortexed for 3 s before the sample was transferred to a 250 μL glass insert placed in an autosampler vial. A volume of 40 μL was injected per run for quantitative analysis by LC-MS/MS. The run time was 6 min.

### 2.4. LC-MS/MS System and Configurations

The HPLC system comprised an Agilent 1100 series gradient pump, degasser, autosampler and column oven (Agilent Technologies, Waldbronn, Germany). The chromatographic separation of analyte, IS and endogenous compounds was performed on an ODS Hypersil C18 column (100 mm × 2.1 mm, 5 μm, Thermo Fisher Scientific, Waltham, Massachusetts, United States), which was preceded by a SecurityGuardTM cartridge (4.0 mm × 3.0 mm, Phenomenex, Torrance, CA, United States). Gradient elution was applied with 0.1% aqueous formic acid (Phase A) and acetonitrile (Phase B). The following gradient program was used: 0–0.1 min: 40% B, 0.1–0.2 min: 40→62% B (linear), 0.2–1.6 min: 62% B, 1.6–1.65 min: 62→40% B (linear), 1.65–6.0 min: 40% B. The flow rate was set at 0.5 mL/min. The column and the autosampler were both maintained at 24 ± 3 °C.

The column eluent was detected by an API 4000 triple quadrupole mass spectrometer (Applied Biosystems, MDS SCIEX, Concord, ON, Canada). The analytes were first nebulized by nitrogen gas, and then introduced into the detector at 500 °C. The optimized entrance potential was 10 V. Nimbolide and exemestane were declustered at 126 V and 60 V respectively, and analyzed by an electropositive ion spray (ESI +ve) of 5500 V. The optimized collision energies were set at 21 V and 33 V for nimbolide and exemestane respectively. The optimized collision cell exit potentials were set at 22 V and 8 V for nimbolide and exemestane respectively. Multiple reaction monitoring (MRM) was employed to monitor the precursor (Q1) and product ion (Q3). The mass spectrometer was tuned to allow the [M + H]^+^ ions of nimbolide (*m*/*z* 467), exemestane (*m*/*z* 297) and paclitaxel (*m*/*z* 854) to pass through the first quadrupole (Q1) and into the collision cell (Q2) for fragmentation. The product ions of nimbolide (*m*/*z* 435), exemestane (*m*/*z* 121) and paclitaxel (*m*/*z* 286) were monitored through the third quadrupole (Q3).

### 2.5. Construction of Standard Curve

The standard calibration curves were constructed using six concentrations. The calibrators were prepared at the following concentrations: 5, 25, 100, 250, 500, and 1000 ng/mL, for nimbolide. Concentrations of nimbolide were back-calculated from the weighted (1/x) linear least squares fitted line of peak area ratio of nimbolide to the IS versus standard concentrations of nimbolide.

### 2.6. Validation Strategy

Validation was performed by establishing intra- and inter-day precision and accuracy of the method on quality controls (QCs). The calibration curves were constructed using six different calibrator concentrations of nimbolide. Intra-day variability was determined by analyzing 4 times the QCs using the same calibration curve. Inter-day variability was determined by analyzing the QCs on four different days using calibration curves obtained daily. The precision of the method at each QC concentration was expressed as a coefficient of variation (CV) by calculating the standard deviation as a percentage of the mean calculated concentration, while the accuracy of the assay was determined by expressing the percentage of the mean from the true value.

### 2.7. Matrix Effect Assessment

The matrix effect is a common and detrimental phenomenon in LC-MS or LC-MS/MS procedures. Per the FDA bio-analytical methods validation guidance for industry, the matrix effect should be investigated to achieve good precision and accuracy.

The matrix effect was investigated by determination of the peak areas of analyte and IS in the matrix-containing tube to those in the reference tubes using post-extraction addition approach. The validation was carried out on QC samples in quadruplicate at each concentration. The concentration levels evaluated were at 15, 300 and 900 ng/mL for nimbolide and 250 ng/mL for IS.

For the matrix-containing tube, 10 μL of blank mouse plasma and 20 μL of methanol were placed in a 1.5 mL PP tube. Thirty microliters of acetonitrile was added subsequently and the PP tube was vortexed for another 30 s. The PP tube was centrifuged at 17,562× *g* for 10 min at 4 °C. Thereafter, 40 μL of the supernatant was transferred to a second 1.5 mL PP tube, followed by the addition of 10 μL of each QC working solution and 10 μL of IS working solution. The sample was dried under nitrogen gas at 50 °C for 45 min. The dried tube was reconstituted with 100 μL of acetonitrile-0.1% formic acid in water (40:60, *v*/*v*). Eighty microliters of the reconstituted mixture was transferred to a 250 μL glass insert in an autosampler vial for analysis. For the reference tube, the procedure was repeated with 10 μL of milli-Q water replacing the blank mouse serum.

### 2.8. Recovery Assessment

Absolute recovery was examined by analyzing the ratios of analyte and IS peak areas in the tube spiked before extraction to those in the tube spiked after extraction. The validation was carried out on QC samples in quadruplicate at each concentration. For the tube spiked before extraction, the steps were carried out per ‘Calibrator and quality control sample preparation’. For the tube spiked after extraction, the steps were carried out as described in ‘Validation strategy’ for the matrix-containing tube.

### 2.9. Stability Assessment

Stability of the analyte in mouse plasma was determined using QC samples in triplicates at each concentration.

#### 2.9.1. Freeze and Thaw Stability

One, three, and six freeze-thaw cycles were selected for stability testing. For each set (consisting of one, three, and six freeze-thaw cycles), three aliquots of each QC concentration were prepared in mouse plasma, stored at −80 °C until completely frozen, and thawed unassisted at room temperature (RT). The freeze-thaw cycle was then repeated for a total of two and five times respectively to execute three and six freeze-thaw cycles. The sample preparation, as described in Section 2.4, with the exception in the initial step in which 10 μL of nimbolide was replaced by 10 μL of methanol, was then carried out to analyze the samples.

#### 2.9.2. Bench-Top Stability

Intervals of 2 and 4 hours were selected for stability testing. Six aliquots of each QC concentration were prepared in mouse plasma and kept on the bench-top. Three aliquots of each QC concentration were taken at each time interval of 2 and 4 h. Sample preparation, as stipulated in calibrator and QC sample preparation, with the exception in the initial step in which 10 μL of nimbolide was replaced by 10 μL of methanol, was then carried out to analyze the samples.

### 2.10. Drug Measurement in Mouse Serum Samples

All procedures involving animals were reviewed and approved by SingHealth Institutional Animal Care and Use Committee (IACUC No is 2015/SHS/1105 which has been approved on 20/10/2015). NCr nude male mice (7–8 weeks old) each weighing 25 g (±2 g) were housed in National Cancer Centre, Singapore, under standard laboratory sterile conditions, and the mice were given ad libitum access to food and water. Eight mice were used for a preliminary PK study at a dose of nimbolide (3 mg/kg) administered via gavage. About 150 µL of blood was taken from facial vein of mice before administration (baseline), and 10, 30, 60, 120, 240, 360 and 480 min post-dosing.

Blood samples were kept for 30 min at RT for clotting, and then centrifuged at 17,562× *g* at 4 °C for 6 min. The serum (supernatant) was transferred into a cryo-vial for storage at −80 °C, and thawed unassisted at RT prior to analysis. Sample preparative procedure was carried out as stipulated in calibrator and QC sample preparation, with the exception in the initial step in which 10 μL of nimbolide was replaced by 10 μL of methanol. The concentration of nimbolide in mouse serum (ng/mL) was derived using interpolation within the standard calibration curve. The unit was then converted to nmol/L via the following equation: Concentration of nimbolide (nmol/L) = (Concentration of nimbolide (ng/mL) × 1000)/466.5.

## 3. Results and Discussion

In recent years, the natural compound nimbolide has attracted considerable research interest owing to its cancer chemotherapeutic and chemopreventive effects in vitro and in vivo [15,16,17,18]. For instance, recent studies have shown that nimbolide potently induced apoptosis in hepatocellular carcinoma cells, and inhibited invasion and migration of breast cancer cells [19,20]. In chemically-induced murine cancer models, nimbolide was shown to simultaneously inhibit phase I carcinogen-activation enzymes (e.g., CYP1A1, CYP1B1) and induce phase II carcinogen-detoxification enzymes (e.g., glutathione-S-transferase, quinone reductase), thereby exhibiting cancer chemopreventive effects by preventing pro-carcinogen activation and oxidative DNA damage [21,22,23]. To our knowledge, this is the first validated LC-MS/MS method for preclinical PK study of nimbolide, as previous preclinical studies have been limited to pharmacodynamic (PD) assessments [24].

Pharmaceutical development from natural products is emerging as a promising strategy for the identification of novel anticancer agents [25,26,27,28,29,30]. Hence, the development of sensitive and robust bio-analytical methods for the quantification of natural anticancer compounds in biological fluids will be tremendously valuable in this regard.

### 3.1. Optimal Selection of Internal Standards

An optimal IS should exhibit highly similar chemical properties to the analyte of interest, but must be distinguishable from the analyte in the mass spectra. Ideally, isotopically-labelled IS should be used for matrix matching used in analysis to compensate for matrix effects that influence analytical response, especially in electro-spray mass spectrometry where ionization suppression is a major problem in accurate quantitative analysis [31], but unfortunately is not always commercially available, particularly for novel or natural compounds, and is often costly.

**Figure 1:**
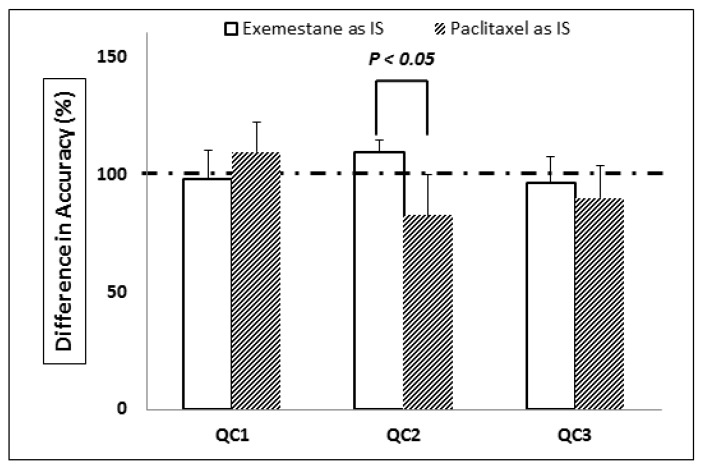
Comparison between accuracies of 3 quality controls of nimbolide in mouse serum using exemestane or paclitaxel as IS.

In the context of this study, isotopically-labelled nimbolide could not be commercially obtained. In addition, no suitable chemical analogues can be used as IS for the determination of nimbolide in LC-MS/MS method. Hence, paclitaxel and exemestane, non-analogues to nimbolide, were selected as the IS candidates because both compounds are available in our laboratory, and have similar Log*P* values to that of nimbolide (Table 1) [32]. The hydrophobicity of an analyte will be the primary indicator of the retentivity in reversed phase HPLC. The molecule with a higher value of Log*P* is more hydrophobic, resulting in longer retention time. Our results demonstrated that the quantitative concentrations of nimbolide in mouse serum with exemestane as IS were more consistent and robust than those with paclitaxel as IS, even though the log*P* value of paclitaxel (2.5) is closer to that of nimbolide (2.2), as compared with the log*P* value of exemestane (3.1). Based on the results of Figure 1, accuracy of 3 QCs with exemestane as IS are within 15%. In contrast, QC2 with paclitaxel as IS showed an accuracy of 82.6%, i.e., that failed to meet the requirements of FDA guidelines.

To investigate underlying reasons for the enhanced suitability of IS for nimbolide, we analyzed important parameters such as the quantity of H-bond acceptors or donors which could lead to fluctuations in mass signal through its potential impact on the ionization process. By comparing the presence of H-bond acceptors or donors in paclitaxel and exemestane with nimbolide, it was found that both nimbolide and exemestane did not possess H-bond donors, whereas paclitaxel had 4 H-bond donors. This result indicated that H-bond donors may be crucial to the optimal selection of a suitable IS. This can be supported by the fact that the QC reproducibility of results is improved significantly when exemestane is adopted as an IS compared with paclitaxel. However, this observation should be further investigated with more compounds with different H-bond donors and acceptors. Taken together, exemestane has been identified as a suitable IS for the development of the LC-MS/MS method for the determination of nimbolide in mouse serum samples, resulting in reliable QC data on inter-day and intra-day accuracy and precision.

### 3.2. Extraction Protocol Optimization

Direct protein precipitation was selected as the optimal method for sample extraction. We chose acetonitrile serum in a volume ratio of 3:1 for final sample preparation method, due to the sufficient sample clean-up as demonstrated in our previous study [26]. Direct protein precipitation is a simple, fast, and cost-effective sample preparation method. Coincidently, Baira et al. just submitted a similar paper regarding quantitative analysis of nimbolide via a LC/MS method in which PPT with cold acetonitrile was used as the protein precipitating agent [34].

### 3.3. Chromatographic and Mass Spectrometric Optimization

**Figure 2:**
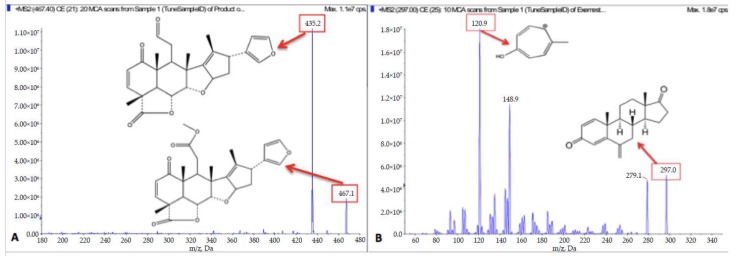
Product ion mass spectra of (**A**) nimbolide at *m*/*z* 467→435 and (**B**) exemestane (IS) at *m*/*z* 297→121.

Product ion mass spectra of nimbolide and exemestane under optimized conditions are shown in Figure 2. A total of three HPLC columns were investigated for chromatographic separation of analytes from endogenous interferences and among analytes. Octadecyl silyl (ODS) Hypersil C18 column, which has suitable hydrophobic characteristics, was chosen as the final chromatographic column due to its acceptable retention and successful baseline separation of nimbolide from the peak of its glucuronide.

We investigated acetonitrile, methanol, and a mixture of acetonitrile/methanol (70:30, *v*/*v*) as organic solvents for the HPLC mobile phase. Acetonitrile was eventually chosen, as it provided shorter run-time and better peak symmetry given the same elution conditions. Aqueous formic acid (0.1%) was chosen as the aqueous solvent due to the increased sensitivity and sharpened peaks. A very low concentration of formic acid was used to avoid undesirable effects on the peak, as it could donate protons and potentially alter the charge of ions.

**Figure 3:**
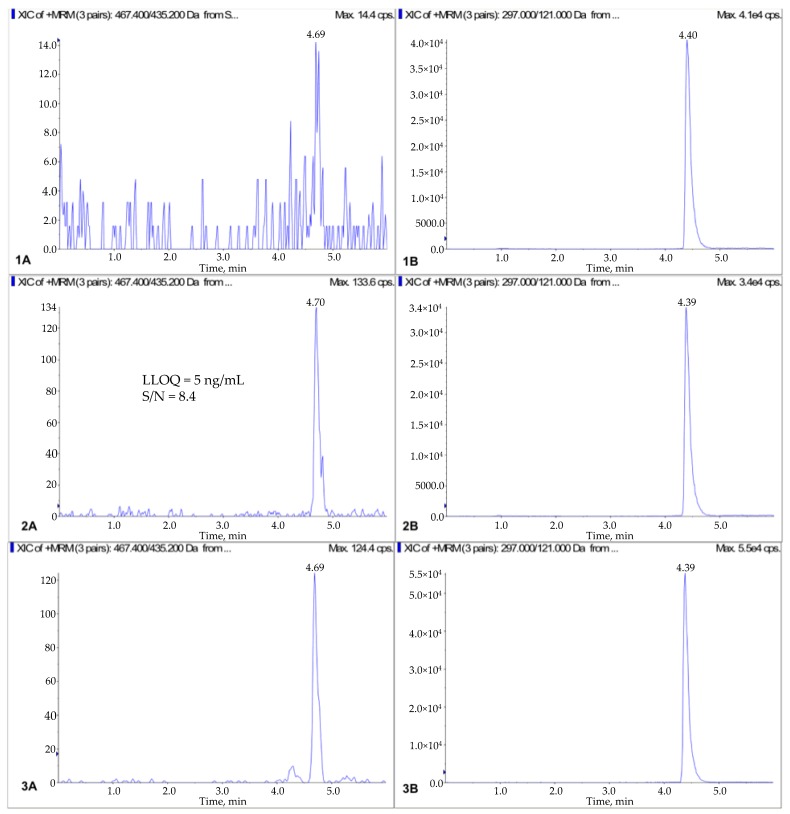
Representative chromatograms of nimbolide (**A**) and IS (**B**): (1) blank plasma, (2) LLOQ (5 ng/mL) in blank plasma, (3) mouse serum taken 8 h after oral administration of 3 mg/kg of nimbolide.

We also investigated isocratic elution and gradient elution programs for chromatographic separation. Gradient elution was ultimately selected, as it resulted in symmetry of the chromatographic peaks for nimbolide and exemestane, and yielded similar retention times: 4.69 and 4.39 min, respectively. Even though there is a 30% difference in hydrophobicity between exemestane and nimbolide, very close retention times were achieved with the help of gradient elution mode, leading to similar elution condition. The chromatograms of standard solution and blank serum are shown in Figure 3.

### 3.4. Selectivity, Carry-Over and Linearity

The selectivity for the optimized method was assessed based on the chromatographic analysis of 6 blank mouse serum samples. The absence of interfering peaks indicated good selectivity under the optimized conditions. No carry-over effect was observed, as the injection of wash following serum with highest spiked concentration showed no peaks at the retention times of nimbolide and exemestane respectively.

The LLOQ was 5.0 ng/mL. Excellent linearity was demonstrated in the range of 5–1000 ng/mL. In the construction of the standard curve, a weighting factor of 1/*x*, where *x* is the concentration, was used to compensate for larger standard deviations of data at higher concentrations, and provide the best fitting curve. The equation of the standard curve is *y* = 0.000714*x* + 0.00132, *r*^2^ = 0.9978, with *y* representing the ratio of nimbolide area to exemestane, and *x* representing plasma concentration of nimbolide.

### 3.5. Accuracy and Precision

The intra- and inter-day precisions for nimbolide were ≤12.6% and ≤13.9% respectively (Table 2), well within the 15% limit set out by FDA guidelines [19].

### 3.6. Matrix Effect

The matrix effect was found to be significant for nimbolide with ion suppression, as the peak area of the analyte present in the matrix was only 32–34% of the nominal concentration (Table 3). The substantial matrix effect of nimbolide could be due to the presence of protein residues and endogenous substances in serum samples. However, the relative matrix effect (expressed as ratio of matrix effect of nimbolide to that of IS) was demonstrated to be consistent at all three concentrations of QC samples, resulting in good linearity. Moreover, the inter-day and intra-day accuracy and precision were consistent, despite the matrix effect.

### 3.7. Recovery

Recovery test results are shown in Table 3. Although the recovery of nimbolide was ~40%, it was consistent and reproducible, thus fulfilling FDA guidelines. Despite its moderate recovery efficiency, direct protein precipitation was still adopted for this study due to its advantages in terms of simplicity and cost-effectiveness.

### 3.8. Stability

Nimbolide stability was found to be within ±15% of nominal concentrations (Table 4), demonstrating that nimbolide was stable in mouse plasma during bench-top storage and freeze-thaw cycles. Our results were also supported by another independent preclinical study in mouse plasma [34].

### 3.9. Application of LC-MS/MS Method

**Figure 4:**
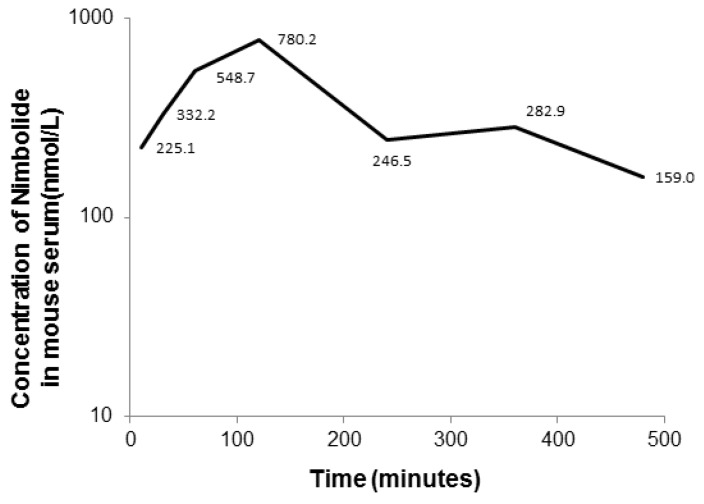
Serum concentration of nimbolide-time profile for mice after oral administration of 3 mg/kg of nimbolide.

In our preclinical PK study, the peak concentration (*C*_max_) of nimbolide after an oral dose of 3 mg/kg was 0.78 µmol/L (Figure 4), which is within the in vitro effective concentration range of 0.5–10 µmol/L, implying that effective therapeutic levels may be achievable in vivo. However, the *C*_max_ was lower than in vitro IC_50_ reported in colorectal and breast cancer [35,36], suggesting that a higher oral dose should be used in in vivo studies of such cancer types. The concentration-time profile indicates that nimbolide is readily and rapidly absorbed orally, and the *C*_max_ occurs ~2 h after oral administration. 

## 4. Conclusions

We have developed a simple, novel, and specific LC-MS/MS method for the quantification of nimbolide in mouse serum using exemestane as the IS. Good linearity was demonstrated within the range of 5–1000 ng/mL. Accuracy and precision were well within FDA guidelines of <15%. The method was also successfully applied for preclinical pharmacokinetic study of nimbolide in mice.

## Figures and Tables

**Table 1:** Comparison of physicochemical properties of nimbolide, exemestane, and paclitaxel [33].

	Nimbolide	Exemestane	Paclitaxel
Molecular weight	466.5	296.4	853.9
Log*P*	2.2	3.1	2.5
H bond acceptors	7	2	15
H bond donors	0	0	4
Chemical structure	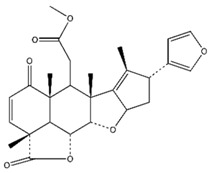	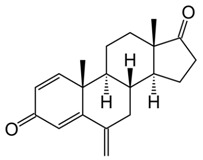	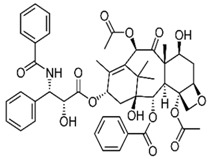

**Table 2:** Intra-day and inter-day precision and accuracy for nimbolide (*n* = 4).

Interval	Nominal Concentration (ng/mL)	Quantified Concentration (Mean ± S.D., ng/mL)	Accuracy (%)	Precision (CV, %)
**Intra-day**	15.0	14.7 ± 1.8	98.2	12.3
	300.0	327.8 ± 15.0	109.3	4.6
	900.0	872.3 ± 109.9	96.9	12.6
**Inter-day**	15.0	14.2 ± 2.0	94.3	13.9
	300.0	330.5 ± 16.7	110.2	5.0
	900.0	934.8 ± 124.5	103.9	13.3

**Table 3:** Matrix effect and recovery of QC samples for nimbolide.

Nominal Concentration (ng/mL)	Matrix Effect on Nimbolide (%)	Matrix Effect on IS (%)	Relative Matrix Effect on Nimbolide	Recovery (%)
15.0	33.7	85.1	0.396	39.0
300.0	32.2		0.378	39.5
900.0	34.3		0.403	39.3

**Table 4:** Bench-top and freeze-thaw stability of QC samples of nimbolide (*n* = 3).

Nominal Concentration (ng/mL)	Stability (Mean ± S.D., %)				
	Bench-Top		Freeze-Thaw		
	2 h	4 h	1 Cycle	3 Cycles	6 Cycles
15.0	108.4 ± 3.4	93.6 ± 4.2	100.5 ± 4.9	86.4 ± 3.7	113.0 ± 7.5
300.0	88.8 ± 2.2	100.1 ± 10.5	101.7 ± 9.4	93.2 ± 12.0	105.1 ± 1.6
900.0	112.2 ± 6.2	101.9 ± 14.5	95.0 ± 5.0	97.3 ± 1.4	107.7 ± 9.4
